# High-Level Fosfomycin Resistance in Vancomycin-Resistant *Enterococcus faecium*

**DOI:** 10.3201/eid2311.171130

**Published:** 2017-11

**Authors:** Yan Guo, Adam D. Tomich, Christi L. McElheny, Vaughn S. Cooper, Amelia Tait-Kamradt, Minggui Wang, Fupin Hu, Louis B. Rice, Nicolas Sluis-Cremer, Yohei Doi

**Affiliations:** University of Pittsburgh Medical Center, Pittsburgh, Pennsylvania, USA (Y. Guo, A.D. Tomich, C.L. McElheny, V.S. Cooper, N. Sluis-Cremer, Y. Doi);; Fudan University Huashan Hospital, Shanghai, China (Y. Guo, M. Wang, F. Hu);; Brown University, Providence, Rhode Island, USA (A. Tait-Kamradt, L.B. Rice);; Fujita Health University, Aichi, Japan (Y. Doi)

**Keywords:** fosfomycin, fosfomycin resistance, vancomycin, vancomycin resistance, Enterococcus faecium, bacteria, vancomycin-resistant enterococci, antimicrobial resistance, UDP-N-acetylglucosamine enolpyruvyl transferase, MurA

## Abstract

Of 890 vancomycin-resistant *Enterococcus faecium* isolates obtained by rectal screening from patients in Pittsburgh, Pennsylvania, USA, 4 had MICs >1,024 μg/mL for fosfomycin. These isolates had a Cys119Asp substitution in the active site of UDP-N-acetylglucosamine enolpyruvyl transferase. This substitution increased the fosfomycin MIC >4-fold and rendered this drug inactive in biochemical assays.

Vancomycin-resistant enterococci can cause nosocomial bacteremia, infective endocarditis, and intraabdominal and urinary tract infections that have limited treatment options. Fosfomycin is an antimicrobial drug that shows a wide spectrum of activity that includes enterococci, staphylococci, and many gram-negative species (*1*). Fosfomycin inactivates UDP-N-acetylglucosamine enolpyruvyl transferase (MurA) by covalent modification of a highly conserved cysteine residue in the active site of MurA (*2*). Some bacterial species, such as *Borrelia burgdorferi* and *Mycobacterium tuberculosis*, are naturally resistant to fosfomycin because they encode an aspartic acid residue instead of cysteine in the active site of MurA. Furthermore, in *Escherichia coli*, substitution of this cysteine at position 115 by aspartic acid results in fosfomycin resistance (*3*).

Fosfomycin has historically shown excellent in vitro activity against vancomycin-resistant enterococci, and therefore might be considered as a treatment option for urinary tract infection caused by this organism (*4*). However, information regarding the activity of fosfomycin against vancomycin-resistant enterococci in the setting of increasing fosfomycin use is limited (*5*). We tested vancomycin-resistant enterococcal isolates obtained from rectal screening cultures at the University of Pittsburgh Medical Center (Pittsburgh, PA, USA) during 2012–2016 for fosfomycin resistance.

## The Study

We tested 890 vancomycin-resistant enterococcal isolates by growth on Mueller-Hinton agar plates containing 100 or 200 μg/mL fosfomycin and 25 μg/mL glucose-6-phosphate (Sigma-Aldrich, St. Louis, MO, USA). Isolates that grew on both selective plates were subjected to determination of MIC by using the agar dilution method on Mueller-Hinton agar plates supplemented with 25 μg/mL glucose-6-phosphate (*6*).

Of 234 isolates that grew on fosfomycin-containing selective plates, MICs were 32 μg/mL for 10 (4.3%), 64 μg/mL for 92 (39.3%), 128 μg/mL for 120 (51.3%), 256 μg/mL for 7 (3.0%), 512 μg/mL for 1 (0.4%), and >1,024 μg/mL for 4 (1.7%). When we used the Clinical and Laboratory Standards Institute breakpoint for urinary tract infections (*6*), we found that 12 isolates (1.3%) had MICs >256 μg/mL and were considered resistant; an additional 120 isolates were considered to have intermediate resistance. The estimated resistance rate of 1.3% is consistent with that reported in a recent surveillance study conducted in the United States (*7*). However, the resistance rate would be much higher if we applied the European Committee on Antimicrobial Susceptibility Testing (Växjö, Sweden) breakpoint of <32 μg/mL for susceptible isolates and >32 μg/mL for resistant isolates.

The 4 vancomycin-resistant enterococci isolates with MICs >1,024 μg/mL were *Enterococcus faecium*. We subjected these isolates and a representative fosfomycin-susceptible *E. faecium* isolate (kindly provided by L. Harrison) to high-throughput paired-end sequencing by using NextSeq (Illumina, San Diego, CA, USA). We performed de novo assembly by using CLC Genomics Workbench version 10.0 (QIAGEN, Valencia, CA, USA). We deposited assembled genome sequences in GenBank (accession nos. SAMN07274321–5).

The 4 fosfomycin-resistant *E. faecium* isolates belonged to sequence type (ST) 17 (n = 2), ST18 (n = 1), and ST233 (n = 1) on the basis of in silico multilocus sequence typing and all had the *vanA* gene. These STs belong to clonal group 17, which is a prominent hospital-adapted vancomycin-resistant *E. faecium* clonal lineage associated with outbreaks in healthcare environments (*8*). None of the isolates had *fosB*, a transferable bacillithiol S-transferase gene associated with fosfomycin resistance (*9*). However, *murA* of the 4 fosfomycin-resistant isolates had a codon change of TGT_Cys119_→GAT_Asp119_ at nucleotide position 355–357, which was not present in the fosfomycin-susceptible control isolate or any of the available *E. faecium* genome sequences and was confirmed by Sanger sequencing (Technical Appendix Figure). The *murA* gene of the 8 remaining fosfomycin-resistant isolates with lower MICs of 256 or 512 μg/mL did not contain the nonsynonymous mutations corresponding to C119D. Therefore, the C119D substitution was specific to isolates with a fosfomycin MIC >1,024 μg/mL.

We amplified wild-type and mutant (C119D) *murA*, with their native promoters, by PCR with primers murA-F-*Eco*RI (5′-GAGA**GAATTC**CATAAAATGAGATGCGGATG-3′) and murA-R-*Bam*HI (5′-GAGA**GGATCC**TTAAGCAATCGTTTGTGCTG-3′) (bold indicates restriction endonuclease site sequences) and cloned them into the shuttle vector pTCV-*lac* (*10*). We selected *E. coli* TOP10 transformants by using kanamycin and erythromycin. After confirmation of sequences, we transformed recombinant plasmids into *E. coli* SM10, subsequently transferred them into *E. faecium* D344S by conjugation, and performed selection by using kanamycin, fusidic acid, and rifampin. The baseline fosfomycin MIC of the host strain was 128 μg/mL. Introduction of pTCV-lac-*murA*^WT^ resulted in a 4-fold increase in the MIC to 512 μg/mL, which might be caused by increased expression of WT MurA produced as a result of complementation. Nonetheless, introduction of pTCV-lac-*murA*^C119D^ yielded a higher MIC of >1,024 μg/mL, which indicated a >4-fold increase in the MIC compared with the *murA*^WT^ control (Technical Appendix Table). This finding provided phenotypic evidence that C119D MurA is less susceptible to inhibition by fosfomycin.

We determined steady-state Michaelis-Menten parameters for recombinant purified wild-type and C119D MurA (Table; Technical Appendix). The C119D substitution in MurA increased the mean ± SD Michaelis constant (*K*_m_ 803.2 ± 180.0 μmol/L) for UDP-N-acetylglucosamine compared with the wild-type enzyme (*K*_m_ 382.8 ± 79.5 μmol/L; p = 0.02), but did not affect the catalytic turnover (*k*_cat_). This increase in *K*_m_ resulted in an ≈2-fold decreased catalytic efficiency (*k*_cat_/*K*_m_) for C119D MurA with respect to UDP-N-acetylglucosamine. In contrast, C119D had no major effect on the kinetic parameters for phosphoenolpyruvate as a substrate (Table). The mean ± SD 50% inhibitory concentration of fosfomycin for wild-type MurA was 176.8 ± 38.3 nmol/L; no inhibition of C119D MurA was observed at concentrations <100 μmol/L fosfomycin (Figure).

**Table:** Michaelis-Menten steady-state kinetic parameters for vancomycin-resistant *Enterococcus faecium* wild-type and C119D MurA*

Enzyme	UNAG (p value)				PEP (p value)			
	*K*_m_, μmol/L	*V*_max_, μmol/L/min	*k*_cat_/min	*k*_cat_/*K*_m_, μmol/L/min	*K*_m_, μmol/L	*V*_max_, μmol/min	*k*_cat_/min	*k*_cat_/*K*_m_, μmol/L/min
WT MurA	382.8 ± 79.5	13.9 ± 1.6	138.7 ± 16.4	0.4	229.0 ± 87.201	29.5 ± 8.4	294.5 ± 83.8	1.3
CD119D MurA	803.2 ± 1,780 (0.02)	11.9 ± 2.2 (NS)	119.4 ± 22.2 (NS)	0.2	304.6 ± 35.2 (NS)	28.6 ± 3.2 (NS)	285.5 ± 32.1 (NS)	0.9

*Values are mean ± SD for >3 independent experiments unless otherwise indicated. Statistical differences between kinetic parameters for vancomycin-resistant enterococci WT and C119D MurA were assessed by using a paired *t*-test. MurA, UDP-N-acetylglucosamine enolpyruvyl transferase; NS, not significant; PEP, phosphoenolpyruvate; UNAG, UDP-N-acetylglucosamine; WT, wild type.

**Figure:**
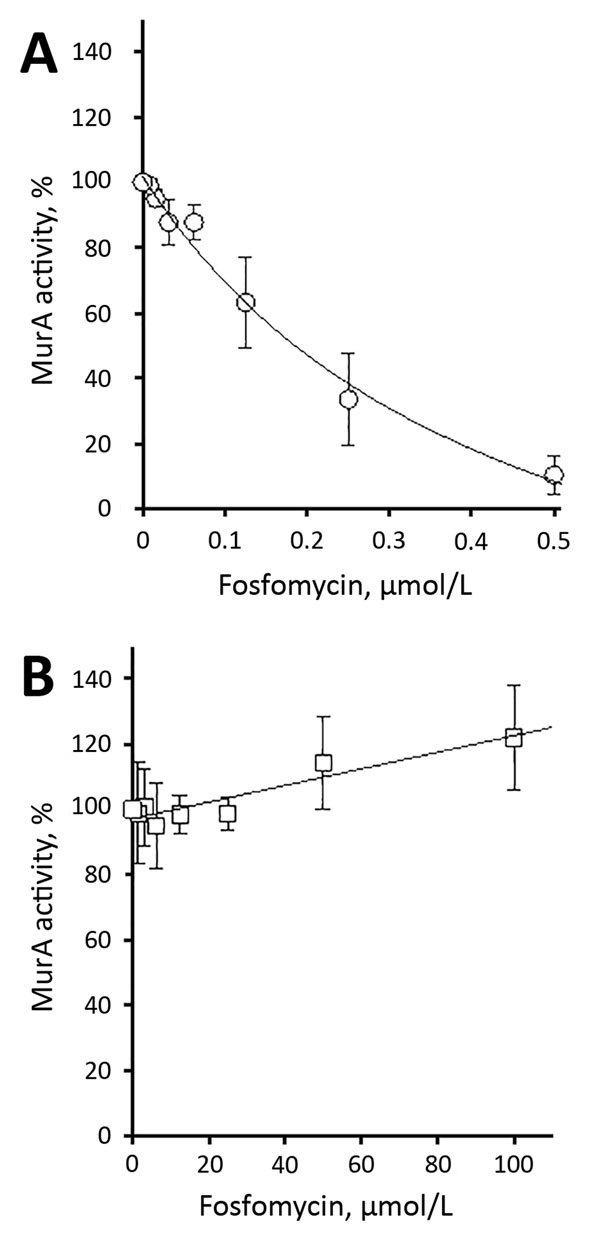
Inhibition of recombinant purified vancomycin-resistant *Enterococcus faecium* wild-type (A) and C119D (B) MurA by fosfomycin. The 50% inhibitory concentration was 176.8 ± 38.3 nmol/L for wild-type MurA and >100 μmol/L for C119D MurA. Error bars indicate mean ± SD of >3 independent experiments. MurA, UDP-N-acetylglucosamine enolpyruvyl transferase.

Our finding that high-level fosfomycin resistance in vancomycin-resistant enterococci can be conferred by substitution of the active site cysteine in MurA is consistent with the mode of action of fosfomycin, which covalently and irreversibly binds to the thiol group of this residue. The MurA enzymes in *M. tuberculosis* and *B. burgdorferi* are refractory to fosfomycin inhibition and naturally possess aspartic acid at the equivalent position (*11*,*12*). Previous site-directed mutagenesis–based studies of *E. coli* MurA showed that aspartic acid and glutamic acid substitutions, although conferring fosfomycin resistance, had a major effect on catalytic functioning of the enzyme (*3*). Specifically, the catalytic efficiency of C115D *E. coli* MurA was reported to be >10-fold less than the wild-type enzyme (*3*).

In our study, the C119D substitution had only minimal effect on vancomycin-resistant enterococci MurA activity. The reason for these differences in kinetic activity is unclear, and additional structure–function studies will be required to elucidate differences between these MurA proteins. Nevertheless, our kinetic data help explain why this substitution was selected in *E. faecium*, whereas *E. coli* –producing C115D MurA has not been identified clinically. The nonsynonymous mutations associated with the C119D substitution of MurA were observed only in isolates that had an MIC >1,024 μg/mL and not in any isolates with lower-level resistance to fosfomycin. Therefore, the mechanisms underlying low-level fosfomycin resistance in enterococci need to be determined.

## Conclusions

In this study, fosfomycin maintained activity against most contemporary vancomycin-resistant enterococci isolates, but we identified high-level resistance caused by substitution of the active site cysteine in MurA, which made it refractory to inhibition by fosfomycin but retained its catalytic activity. Our finding that high-level resistance to fosfomycin might arise through mutations of the target enzyme MurA, accompanied by modest impairment of the catalytic activity, indicates the need for ongoing surveillance activities to ensure its activity against vancomycin-resistant enterococci is maintained. In addition, this finding highlights the potential relevance of aspartic acid−substituted, catalytically active MurA enzymes as a target for inhibitor development.

Technical Appendix Additional information on high-level fosfomycin resistance in vancomycin-resistant *Enterococcus faecium*.
