# Supplementary material for: High-Level Fosfomycin Resistance in Vancomycin-Resistant Enterococcus faecium
Source: Emerg Infect Dis. 2017 Nov;23(11):1902–4. doi: 10.3201/eid2311.171130 (PMC5652432; doi:10.3201/eid2311.171130)
Supplement: Technical Appendix — Additional information on high-level fosfomycin resistance in vancomycin-resistant Enterococcus faecium. [file 17-1130-Techapp-s1.pdf]

Article DOI: https://doi.org/10.3201/eid2311.171130

# High-Level Fosfomycin Resistance in Vancomycin-Resistant *Enterococcus faecium*

## Technical Appendix

## Supplementary Methods

### Cloning and Purification of MurA

Wild-type and C119D UDP-N-acetylglucosamine enolpyruvyl transferase (*murA*) genes were synthesized (Genscript, Piscataway, NJ, USA), cloned into the pE-SUMOstar prokaryotic expression vector (LifeSensors, Malvern, PA, USA), and transformed into *Escherichia coli* BL21 (DE3) pLysS competent cells (Promega, Madison, WI, USA). Transformed *E. coli* BL21 (DE3) were grown overnight at 37°C in Power Prime Broth (AthenaES, Baltimore, MD, USA) containing 100 mg/L of ampicillin. Overnight cultures were diluted 1:50 in fresh Power Prime Broth, grown to midlog phase (optical density = 0.3 at 600 nm), before protein expression was induced for 4 h at 37°C by addition of 1 mmol/L isopropyl β-D-1-thiogalactopyranoside. Cells were harvested by centrifugation, suspended in 50 mmol/L sodium phosphate buffer (pH 7.8) containing protease inhibitor (lysis buffer), and lysed by using a French press.

Cell supernatants were mixed with prepared TALON Metal Affinity Resin (Clontech Laboratories, Inc., Mountain View, CA, USA), loaded onto a gravity-flow column, and washed with 50 mmol/L sodium phosphate (pH 7.8) containing 0.3 mol/L NaCl and 1 mmol/L β-mercaptoethanol. Bound protein was eluted with 100 mmol/L sodium phosphate (pH 6.0) containing 0.6 mol/L NaCl, 240 mmol/L imidazole, and 1 mmol/L β-mercaptoethanol. The protein was then exchanged into 25 mmol/L Tris-HCl (pH 7.5) by using an NAP-25 column (GE Healthcare, Chicago, IL, USA). Protein concentration was determined by using a Bradford assay with bovine serum albumin as the standard. Purity was assessed by using sodium dodecyl sulfate–polyacrylamide gel electrophoresis. Purified proteins were stored in 25% glycerol at –80°C.

### MurA Steady-State Kinetic Assays

MurA (100 nmol/L) was incubated with various concentrations of UDP-N-acetylglucosamine (UNAG) (0–3 mmol/L) or phosphoenolpyruvate (PEP) (0–1 mmol/L) in 50 mmol/L Tris-HCl buffer (pH 7.5) at 37°C for 10 min. Reactions were initiated with 300 µmol/L PEP (for various concentrations of UNAG) or 3 mmol/L UNAG (for various concentrations of PEP) at 37°C for 20 min. Reactions were then quenched, and inorganic phosphate was quantified by using the Malachite Green Phosphate Assay Kit, (BioAssay Systems, Hayward, CA, USA) per the manufacturer’s recommendations. Data were fitted to Michaelis-Menten equations by using GraphPad Prism version 6 (GraphPad Software, San Diego, CA, USA).

### Fosfomycin Inhibition Assay

For inhibition assays, 100 nmol/L of wild-type or mutant MurA was incubated with various concentrations of fosfomycin and 3 mmol/L UNAG at 37°C for 10 min. Reactions were initiated with 300 µmol/L PEP, and inorganic phosphate was quantified as described. The concentration of fosfomycin that resulted in 50% inhibition was determined by using GraphPad Prism version 6.

**Technical Appendix Table.** Characteristics of strains and plasmids used in the study of high-level fosfomycin resistance in vancomycin-resistant *Enterococcus faecium**

| Strain or plasmid | Fosfomycin MIC, mg/L | Description/use |
|---|---|---|
| Clinical strain | | |
| *Enterococcus faecium* 2014–7 | >1,024 | *murA*$^{C119D}$ |
| *E. faecium* 2014–195 | >1,024 | *murA*$^{C119D}$ |
| *E. faecium* 2015–149 | >1,024 | *murA*$^{C119D}$ |
| *E. faecium* 2016–78 | >1,024 | *murA*$^{C119D}$ |
| *E. faecium* 2016–194 | 64 | *murA*$^{WT}$ |
| Transformant | NA | NA |
| *E. faecium* D344S (pTCV-lac$^{C119D}$) | >1,024 | *murA*$^{C119D}$ |
| *E. faecium* D344S (pTCV-lac$^{WT}$) | 512 | *murA*$^{WT}$ |
| *E. faecium* D344S (pTCV-lac) | 128 | Control |
| Host strain | NA | NA |
| *E. faecium* D344S | NA | Plasmid transfer |
| *Escherichia coli* SM10 | NA | Plasmid transfer |
| *E. coli* TOP10 | NA | Plasmid transfer |
| *E. coli* BL21 (DE3) | NA | Protein expression |
| Plasmid | NA | NA |
| pTCV-lac | NA | Cloning of *murA*; erythromycin and kanamycin resistant |
| pE-SUMOstar | NA | Expression of *murA*; ampicillin resistant |

*murA, UDP-N-acetylglucosamine enolpyruvyl transferase; NA, not applicable; WT, wild type.

```
MurA_2014-7         MEEIIVRGGNQLNGTVRIEGAKNAVLPILAASLLAEEGITTLDNVPILSDVFTMNQVIRH 60
MurA_2014-195       MEEIIVRGGNQLNGTVRIEGAKNAVLPILAASLLAEEGITTLDNVPILSDVFTMNQVIRH
MurA_2015-149       MEEIIVRGGNQLNGTVRIEGAKNAVLPILAASLLAEEGITTLDNVPILSDVFTMNQVIRH
MurA_2016-78        MEEIIVRGGNQLNGTVRIEGAKNAVLPILAASLLAEEGITTLDNVPILSDVFTMNQVIRH
MurA_2016-194       MEEIIVRGGNQLNGTVRIEGAKNAVLPILAASLLAEEGITTLDNVPILSDVFTMNQVIRH
MurA_consensus      MEEIIVRGGNQLNGTVRIEGAKNAVLPILAASLLAEEGITTLDNVPILSDVFTMNQVIRH
                    ************************************************************

MurA_2014-7         LNVDVDFDEQKNQVTIDASRQLEIEAPYEYVSQMRASIVVMGPLLARNGHAKVAMPGGDA 120
MurA_2014-195       LNVDVDFDEQKNQVTIDASRQLEIEAPYEYVSQMRASIVVMGPLLARNGHAKVAMPGGDA
MurA_2015-149       LNVDVDFDEQKNQVTIDASRQLEIEAPYEYVSQMRASIVVMGPLLARNGHAKVAMPGGDA
MurA_2016-78        LNVDVDFDEQKNQVTIDASRQLEIEAPYEYVSQMRASIVVMGPLLARNGHAKVAMPGGDA
MurA_2016-194       LNVDVDFDEQKNQVTIDASRQLEIEAPYEYVSQMRASIVVMGPLLARNGHAKVAMPGGCA
MurA_consensus      LNVDVDFDEQKNQVTIDASRQLEIEAPYEYVSQMRASIVVMGPLLARNGHAKVAMPGGCA
                    ********************************************************** *

MurA_2014-7         IGKRPIDLHLKGFQALGAKIIQKNGYIEAIADELIGNTIYLDFPSVGATQNIMMAAVKAK 180
MurA_2014-195       IGKRPIDLHLKGFQALGAKIIQKNGYIEAIADELIGNTIYLDFPSVGATQNIMMAAVKAK
MurA_2015-149       IGKRPIDLHLKGFQALGAKIIQKNGYIEAIADELIGNTIYLDFPSVGATQNIMMAAVKAK
MurA_2016-78        IGKRPIDLHLKGFQALGAKIIQKNGYIEAIADELIGNTIYLDFPSVGATQNIMMAAVKAK
MurA_2016-194       IGKRPIDLHLKGFQALGAKIIQKNGYIEAIADELIGNTIYLDFPSVGATQNIMMAAVKAK
MurA_consensus      IGKRPIDLHLKGFQALGAKIIQKNGYIEAIADELIGNTIYLDFPSVGATQNIMMAAVKAK
                    ************************************************************

MurA_2014-7         GTTIIENVAREPEIVDLANILNKMGAQVYGAGTETMRIEGVDHLHAVNHSIVQDRIEAGT 240
MurA_2014-195       GTTIIENVAREPEIVDLANILNKMGAQVYGAGTETMRIEGVDHLHAVNHSIVQDRIEAGT
MurA_2015-149       GTTIIENVAREPEIVDLANILNKMGAQVYGAGTETMRIEGVDHLHAVNHSIVQDRIEAGT
MurA_2016-78        GTTIIENVAREPEIVDLANILNKMGAQVYGAGTETMRIEGVDHLHAVNHSIVQDRIEAGT
MurA_2016-194       GTTIIENVAREPEIVDLANILNKMGAQVYGAGTETMRIEGVDHLHAVNHSIVQDRIEAGT
MurA_consensus      GTTIIENVAREPEIVDLANILNKMGAQVYGAGTETMRIEGVDHLHAVNHSIVQDRIEAGT
                    ************************************************************

MurA_2014-7         FMVAAAMTQGNVLIADAISEHNRPLISKLIEMGAEIIEEEGGVRVIGPKHILPTDVKTMP 300
MurA_2014-195       FMVAAAMTQGNVLIADAISEHNRPLISKLIEMGAEIIEEEGGVRVIGPKHILPTDVKTMP
MurA_2015-149       FMVAAAMTQGNVLIADAISEHNRPLISKLIEMGAEIIEEEGGVRVIGPKHILPTDVKTMP
MurA_2016-78        FMVAAAMTQGNVLIADAISEHNRPLISKLIEMGAEIIEEEGGVRVIGPKHILPTDVKTMP
MurA_2016-194       FMVAAAMTQGNVLIADAISEHNRPLISKLIEMGAEIIEEEGGVRVIGPKHILPTDVKTMP
MurA_consensus      FMVAAAMTQGNVLIADAISEHNRPLISKLIEMGAEIIEEEGGVRVIGPKHILPTDVKTMP
                    ************************************************************

MurA_2014-7         HPGFPTDMQAQMTAIQLVAEGTSVVTETVFENRFQHLEEMRRMNAHVKIDGNVAIMDGNH 360
MurA_2014-195       HPGFPTDMQAQMTAIQLVAEGTSVVTETVFENRFQHLEEMRRMNAHVKIDGNVAIMDGNH
MurA_2015-149       HPGFPTDMQAQMTAIQLVAEGTSVVTETVFENRFQHLEEMRRMNAHVKIDGNVAIMDGNH
MurA_2016-78        HPGFPTDMQAQMTAIQLVAEGTSVVTETVFENRFQHLEEMRRMNAHVKIDGNVAIMDGNH
MurA_2016-194       HPGFPTDMQAQMTAIQLVAEGTSVVTETVFENRFQHLEEMRRMNAHVKIDGNVAIMDGNH
MurA_consensus      HPGFPTDMQAQMTAIQLVAEGTSVVTETVFENRFQHLEEMRRMNAHVKIDGNVAIMDGNH
                    ************************************************************

MurA_2014-7         ELQGAEVYATDLRAAAALVLAGLKANGITRVRNLNYLDRGYYNFHIKLQQLGADVERVDM 420
MurA_2014-195       ELQGAEVYATDLRAAAALVLAGLKANGITRVRNLNYLDRGYYNFHIKLQQLGADVERVDM
MurA_2015-149       ELQGAEVYATDLRAAAALVLAGLKANGITRVRNLNYLDRGYYNFHIKLQQLGADVERVDM
MurA_2016-78        ELQGAEVYATDLRAAAALVLAGLKANGITRVRNLNYLDRGYYNFHIKLQQLGADVERVDM
MurA_2016-194       ELQGAEVYATDLRAAAALVLAGLKANGITRVRNLNYLDRGYYNFHIKLQQLGADVERVDM
MurA_consensus      ELQGAEVYATDLRAAAALVLAGLKANGITRVRNLNYLDRGYYNFHIKLQQLGADVERVDM
                    ************************************************************

MurA_2014-7         DQTSAEKTAQTIA 433
MurA_2014-195       DQTSAEKTAQTIA
MurA_2015-149       DQTSAEKTAQTIA
MurA_2016-78        DQTSAEKTAQTIA
MurA_2016-194       DQTSAEKTAQTIA
MurA_consensus      DQTSAEKTAQTIA
                    *************
```

**Technical Appendix Figure.** Alignment of the deduced amino acid sequences of wild type and C119D UDP-N-acetylglucosamine enolpyruvyl transferase. (MurA) of vancomycin-resistant *E. faecium* isolates. Strains 2014–7, 2014–195, 2015–149, and 2016–78 have fosfomycin MICs >1,024 mg/L. Strain 2016–194 has a fosfomycin MIC of 64 mg/L. The consensus sequence is derived from WP_002289003.1 and represents the MurA sequence of 450 *E. faecium* strains available in GenBank. Box indicates change from cysteine to aspartic acid at position 119. Asterisks indicate identity at each position.
